# Clinical efficacy of soft tissue micro-adjustment combined with traction in pediatric atlantoaxial subluxation: a randomised controlled study protocol using musculoskeletal ultrasound technology

**DOI:** 10.3389/fped.2025.1696802

**Published:** 2025-11-10

**Authors:** Shuaizi Yin, Huasong Luo, Yinfeng Guo, Ge Cai, Ting Wu, Shuaiyu Ying, Xiao Zhang, Yi Sun, Xiayang Zeng

**Affiliations:** 1 Tui Na Department, Hangzhou Hospital of Traditional Chinese Medicine, Hangzhou TCM Affiliated to Zhejiang Chinese Medicine University, Hangzhou, China; 2 Tui Na Department, Zhejiang Hospital, Hangzhou, China

**Keywords:** atlanto-axial rotatory subluxation (AARS), musculoskeletal ultrasound technology(MUSU), soft tissue, micro-adjustment, randomized controlled trial

## Abstract

**Background:**

Atlanto-axial rotatory subluxation (AARS) in pediatric patients is characterized by abnormal or restricted motion between the atlas and axis vertebrae, typically presenting with neck pain, limited mobility, torticollis, and muscle stiffness. Cervical atlanto-occipital joint x-ray in open-mouth position or CT scan reveals an atlanto-occipital distance (AOD) of 2 mm < AOD < 5 mm, or a bilateral atlanto-occipital lateral distance (B-LAD) ≥ 2 mm. Although traditional manipulative and bone-setting techniques in Traditional Chinese Medicine have demonstrated clinical benefits, robust empirical evidence remains limited. Musculoskeletal ultrasound (MUSU), as a modern diagnostic modality, has gained popularity for assessing musculoskeletal disorders.

**Methods:**

This study utilized a randomized controlled trial design, where eligible patients diagnosed with AARS were, randomly assigned (1:1) to either a treatment group (gentle manipulative technique combined with continuous traction) or a control group (traction plus cervical collar immobilization). The treatment group received treatment twice daily, five days per week, for two weeks. The control group received the same traction protocol but wore a neck brace immediately after each traction treatment. The primary outcome measure was musculoskeletal ultrasound (MUSU) findings, while secondary outcomes include Visual Analogue Scale (VAS) pain scores and Neck Disability Index (NDI) scores. Both groups were assessed at baseline, post-treatment, two weeks, and six months after treatment.

**Discussion:**

The primary objective of this study aims to quantitatively assess the efficacy and safety of gentle manipulative therapy combined with continuous traction in pediatric AARS, using MUSU technology.

**Clinical Trial Registration**: http://itmctr.ccebtcm.org.cn/, identifier ITMCTR2025001572.

## Background

Atlanto-axial rotatory subluxation (AARS) in children refers to impaired or fixed motion between the atlas and axis vertebrae of the cervical spine (1), often caused by factors such as upper respiratory infection, cervical inflammation, poor posture, or neck strains (2). Atlanto-axial rotatory subluxation (AARS) typically presents with symptoms like cervical pain, restricted movement, head and neck tilt, and muscle stiffness in the neck muscles (3). Accompanied by torticollis, vertigo, ataxia, and even complications such as stroke (4). Research indicates that AARS is quite relatively common in pediatric populations, accounting for approximately 70% of spinal disorders in children, with the highest rates seen in those under 12 years old, where the incidence is estimated at 68% (5). In contrast, the occurrence of AARS in adults is around 16% (6). If not treated promptly and effectively, the symptoms can persist or recur, negatively impacting the child's physical appearance and quality of life and increasing psychological stress. In severe cases, it may lead to spinal cord compression, which could result in paralysis or other life-threatening conditions. This predicament imposes considerable emotional and financial stresses on families and society at large.

The concept of atlanto-axial rotatory subluxation (AARS) was first introduced by Bell in 1830, yet nearly two centuries later, our understanding of this condition remains insufficient both domestically and internationally (3). The terminology itself is still debated, with synonyms such as Grisel's syndrome, atlantoaxial subluxation, and spontaneous atlanto-axial subluxation being used interchangeably (7). However, most researchers prefer the term “atlanto-axial rotatory subluxation,” which more accurately describes the underlying pathological characteristics and distinguishes the condition from treatments for atlanto-axial dislocation (8). From a Western medical viewpoint, the causes of AARS are seen as complex and varied, with mechanisms that are not fully understood. Some researchers classify the causes into traumatic and non-traumatic categories, while others break them down further into traumatic, spontaneous, congenital, pathological, degenerative, metabolic conditions, and various induced factors (9, 10). In terms of cervical spine biomechanics, changes in cervical curvature have been associated with atlantoaxial instability. From a biomechanical perspective, there are differences between the cervical spine of children and that of adults. The differences are primarily manifest in increased spinal elasticity, greater ligamentous laxity, shallower horizontal articular processes, and the presence of unossified cartilage in children (11). It has been demonstrated that enhanced spinal elasticity in children is associated with increased spinal mobility, but concomitantly reduced stability (12). In addition, heightened ligamentous laxity has been shown to elevate the risk of atlantoaxial joint instability (12). Furthermore, the cartilage in children differs from that in adults in that it has not yet ossified, thereby further increasing the risk of instability and susceptibility to infection. In summary, from a biomechanical perspective, the atlantoaxial joint in children exhibits instability, rendering it more susceptible to subluxation and dislocation. Additionally, some researchers have conducted statistical analyses of clinical data, finding correlations with upper respiratory infections, localized cervical inflammation, and surgical procedures involving the head and neck (13).

Currently, therapeutic approaches for pediatric atlanto-axial rotatory subluxation (AARS) can be broadly divided into two main categories: conservative and surgical interventions (14). Conservative strategies mainly involve the use of non-steroidal anti-inflammatory drugs (NSAIDs), cervical collar traction, and skull traction combined with plaster fixation (15, 16). Surgical options primarily focus on anatomical realignment through atlanto-axial fusion procedures, which include techniques such as Gallie, Harms, and Magerl fusion (17). However, conservative methods like oral medications and traction therapies may have their limitations; including concerns about drug toxicity and the risk of recurrence (18). Furthermore, both conservative and surgical interventions carry the potential for surgical trauma, and may affect the mobility of the cervical spine, potentially leading to various postoperative complications (5).

In Traditional Chinese Medicine (TCM), there is a core belief that muscles should remain flexible, be properly positioned, and respond well to warmth while resisting cold (19). It is thought that muscles flourish in a soft environment and suffer from rigidity; additionally, a good blood supply is vital for muscle health. When blood circulation is smooth, muscle function improves (20). The relationship between muscles and bones is essential. When bones are aligned correctly, muscles can be more flexible. Flexible muscles help maintain bone alignment (21). Muscle contractions and extensions enable bone movements. Bones provide the necessary support for various bodily movements. Ideally, a state of balance, known as “muscle-bone balance,” exists between muscles and bones (22). In children, a condition referred to as AARS is understood as “muscle misalignment and bone misalignment,” which is manifested as reduced muscle flexibility, disordered muscle positioning, and skeletal misalignment (23).

Currently, there are various Traditional Chinese Medicine (TCM) treatment methods available to pediatric atlanto-axial rotatory subluxation (AARS), all of which have been validated through clinical practice. Among these methods, manipulation, bone-setting, and traction are considered the most effective (24, 25). Manipulative techniques work by improving blood circulation in the cervical and shoulder areas through thermal effects, which enhance the elasticity of muscle contractions (26). These techniques also stimulate muscle stretch receptors, making them more sensitive; this indirectly activates the sensory cortex and suppresses the reticular formation in the brainstem. This series of effects leads to reduced local muscle tension and relief from muscle spasms in the cervical and shoulder regions, thereby promoting greater muscular flexibility. Techniques focused on muscle relaxation and restoration can quickly alleviate symptoms such as dizziness, neck pain, and muscle spasms or tenderness that are associated with atlanto-axial joint disorders. TCM bone-setting methods utilize leverage principles, often using the jaw or the side of the head as a pivot point (27). By applying a rotational force known as “inch force”, practitioners can transmit force through the cranio-atlanto-axial joint to realign the axis vertebra, achieving what is referred to as “bone alignment”, which specifically means restoring the proper anatomical position of the vertebra to improve joint function and reduce symptoms. This process effectively minimizes the negative impacts of skeletal misalignment on surrounding tissues, thereby promoting a healing response (28).

The gentle muscle adjustment technique used with continuous traction is based on the idea of balancing attention between muscles and bones, prioritizing muscle relaxation to aid in bone alignment. By applying continuous traction while the patient lies on their back, the therapist can make adjustments to the traction direction based on the child's neck discomfort and tilt. This creates a localized mechanical dynamic condition in the cervical area. This method is specifically designed to accommodate the child's muscle condition, helping to ease discomfort and improve the child's cooperation during treatment, which in turn promotes relaxation. This relaxation helps reduce neck muscle tension, alleviates nerve root compression, and improves symptoms such as neck pain and head tilt. To build on this, gentle muscle techniques are employed. Pressing, kneading, pinching, and rubbing are used to stimulate overactive muscle groups, including the sternocleidomastoid, trapezius, and scalene muscles. These techniques enhance local blood flow and energy circulation, relieve muscle spasms and pain, restore muscle elasticity, and reduce local adhesions, thereby making the muscles more flexible. With this improved muscle flexibility, passive, active, and specific neck movements can be performed, along with engaging in relaxed and pleasant communication with the patient. This allows for effective mechanical traction of the neck and shoulder muscles in a dynamic state, helping to realign misaligned bones and achieve proper bone alignment, ultimately harmonizing the muscular and skeletal components of the atlanto-axial joint.

Currently, therapeutic strategies for pediatric AARS primarily focus on either balancing the treatment of muscles and bones or prioritizing skeletal alignment over muscular interventions. Clinical efficacy assessments largely depend on subjective evaluations or measurements of bone structure. There is a pressing need for thorough investigations into gentle muscle-oriented treatment regimens for pediatric AARS. This highlights the importance of designing methodologically rigorous randomized controlled trials and studies that include objective observational indicators focused on gentle muscle-oriented techniques. Such rigorous research efforts would provide a crucial foundation for standardizing conservative therapeutic approaches for pediatric AARS.

Musculoskeletal ultrasound (MUSU) is an innovative diagnostic technique that offers numerous benefits, such as being cost-effective, user-friendly, quick to perform, and capable of dynamic evaluations (29). It is widely used for diagnosing and screening musculoskeletal disorders (30, 31). One of its advanced applications is real-time shear wave elastography (SWE), a non-invasive imaging method that evaluates the mechanical properties of soft tissues by measuring the transient deformations and displacements caused by ultrasound waves. Shear waves are transverse waves that propagate through tissues, and their speed reflects the tissue's stiffness (32). The elasticity of these tissues indicates their mechanical hardness, and ultrasound elastography provides an objective way to quantify this characteristic, often using parameters like shear modulus or Young's modulus to represent muscle stiffness. There is a direct relationship where increased tissue hardness correlates with faster shear wave propagation, higher Young's modulus values, and reduced tissue elasticity (33). Research has shown a notable increase in the hardness of muscle groups, including the trapezius, scalene, and sternocleidomastoid muscles, in pediatric patients with Adolescent Idiopathic Scoliosis (AARS). This finding underscores the crucial role of MUSU technology in objectively assessing changes in muscle morphology and elasticity in this area of research (34).

This investigation employs a gentle muscle adjustment technique combined with continuous traction as a therapeutic intervention for pediatric patients diagnosed with AARS. A clinical randomized controlled trial design is implemented to assess changes in Visual Analog Scale (VAS) pain scores and Neck Disability Index (NDI) scores, as well as alterations in muscle morphology and elasticity before and after treatment. By quantitatively evaluating the clinical effectiveness of the gentle muscle adjustment technique under continuous traction through MUSU, this study aims to elucidate the specific clinical benefits of this treatment for pediatric AARS. Consequently, it seeks to promote the broader application of this therapeutic approach in the future.

## Methods

### Objective

The objective of this investigation is to clarify the effectiveness and safety of the gentle muscle adjustment technique combined with continuous traction in treating pediatric AARS. We aim to develop a treatment protocol that is not only effective and straightforward but also safe and cost-effective. Furthermore, we will evaluate the role of MUSU technology in assessing changes in neck muscle morphology and elasticity, thereby providing quantitative evidence to support conservative management strategies for pediatric AARS.

### Trial design

**Figure 1:**
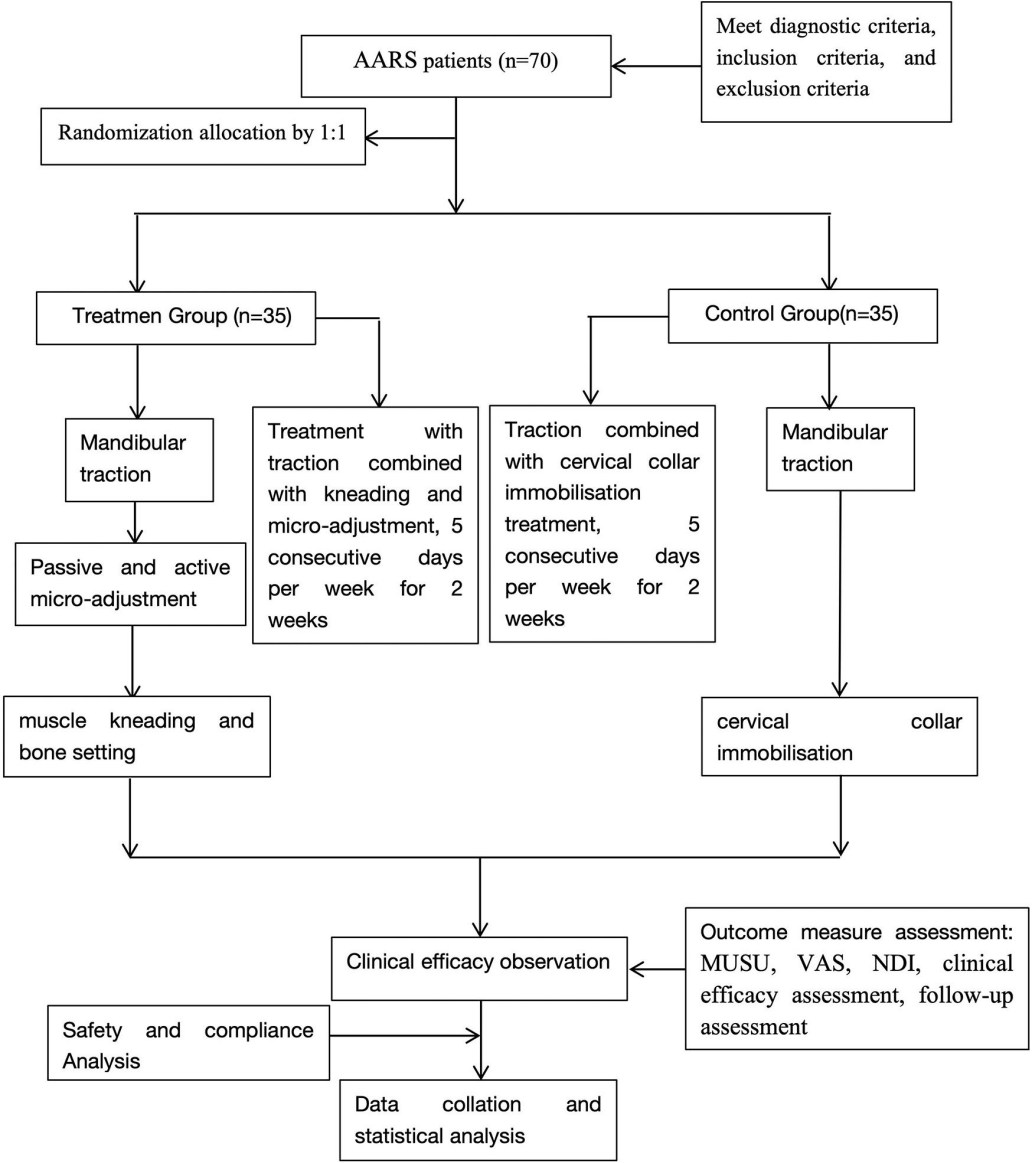
Flow chart of the study process. AARS, atlanto-axial rotatory subluxation; MUSU, Musculoskeletal ultrasound; VAS, Visual Analog Scale; NDI, Neck Disability Index.

**Figure 2:**
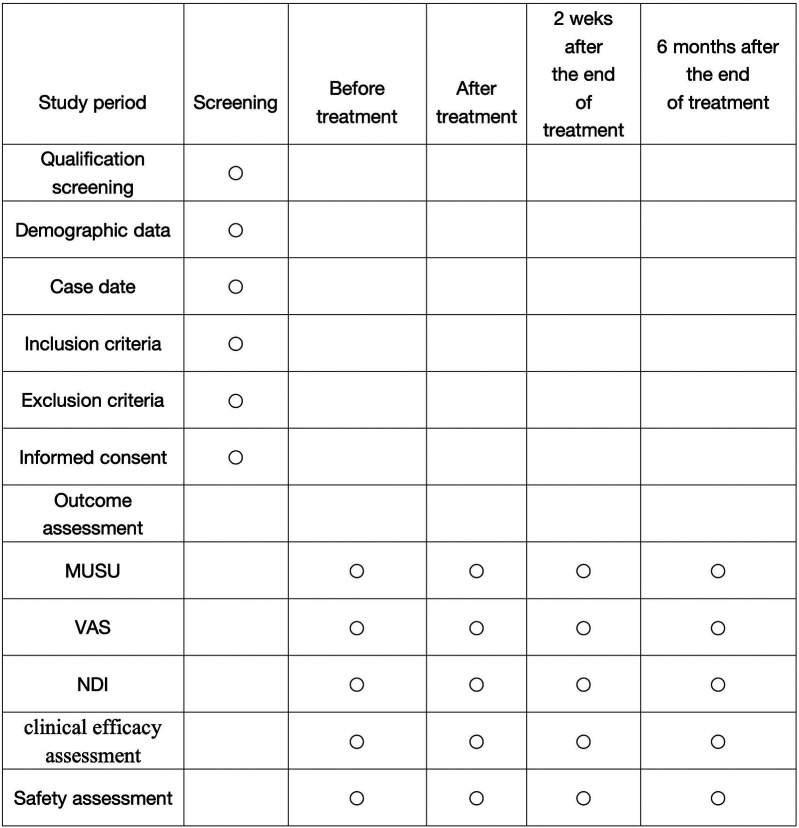
Schedule of enrolment, treatments, and assessments. MUSU, Musculoskeletal ultrasound; VAS, Visual Analog Scale; NDI, Neck Disability Index.

This research utilizes a single center randomized, controlled design. Due to the specific nature of the treatment protocols, it is not feasible to blind either the researchers or the participants. Therefore, the study adopts a blind evaluation approach. Under this approach, an independent third-party conducts assessments using various scales, ultrasound examinations, and statistical analyses. This method ensures a clear “three-way separation” among researchers, operators, and randomly assigned in a 1:1 ratio to either a treatment group (gentle muscle adjustment with continuous traction) or a control group (traction combined with cervical-collar fixation for patients with AARS. We expect to randomly assign 70 patients diagnosed with AARS to participate in this study. Randomization will be computer-generated by a statistician independent of the research team. The trial protocol has been registered on the International Traditional Medicine Clinical Trial Registry (http://itmctr.ccebtcm.org.cn/) which is accredited by the World Health Organization as a Level 1 registry, the Clinical trial number: NO. ITMCTR2025001572, the registration date is 19 August 2025, and the report adheres strictly to the guiding principles outlined in the relevant guidelines. Figure 1 provides a flowchart that details the research process. Furthermore, the schedule for the enrollment, treatment and assessment of the trial is show in Figure 2.

### Participants, recruitment, and ethical considerations

The recruitment of participants will be recruited through two main channels: printed recruitment posters within the hospital and online outreach through affiliated networks. During recruitment, potential participants and their guardians will receive detailed information about the inclusion and exclusion criteria, intervention methods, study duration, and potential benefits. After confirming eligibility, informed consent will be obtained from the child's legal guardian. Once consent is documented, participants will be randomly assigned to one of the study groups.

The study protocol has been approved by the Ethics Committee of Hangzhou Traditional Chinese Medicine Hospital, (2024KLL093).

### Diagnostic criteria

The diagnostic criteria for pediatric atlantoaxial rotatory subluxation are established based on the criteria outlined in Xu Shaoting's edition of “Practical Orthopedics”: These criteria include several key components: (1) the patient's history should reveal a recent upper respiratory infection, neck infection, or poor neck posture; (2) clinical symptoms may present as neck pain and neck tilt, with more severe cases which may lead to dizziness and nausea; (3) physical signs can include limitations in cervical movement—particularly restricted flexion, extension, and rotation—accompanied by neck muscle stiffness, asymmetrical muscle development, tenderness at the posterior transverse process of the axis, or visible displacement; and (4) Radiological assessments, such as x-ray or CT demonstrating an atlantodental interval (ADI) falls within the range of 2 mm–5 mm. Additionally, there may be a lateral atlanto-dental interval difference (VBLADS) of 2 mm or greater.

### Inclusion criteria

Participants must meet the following inclusion criteria: (1) fulfillment of the diagnostic criteria for atlantoaxial rotatory subluxation (AARS); (2) age between 6 and 14 years, regardless of sex; (3) voluntary participation with informed consent provided by the child's guardian; and (4) first episode of the condition with no treatment received within the preceding two weeks. All participants must commit to completing the entire two-week treatment protocol and follow-up assessments.

### Exclusion criteria

Participants will be excluded if they meet any of the following criteria: (1) a history of severe trauma resulting in fractures or complete dislocation of the atlanto-axial joint, or congenital abnormalities of the joint; (2) diagnosis of muscular torticollis or any other condition not consistent with the diagnostic criteria for AARS; (3) severe cardiopulmonary disease, history of head or neck surgery, or psychiatric disorders; (4) inability to cooperate with treatment or assessment procedures due to physical or cognitive impairments; or (5) any medical condition rendering participation unsafe or impractical according to the investigator's judgment.

### Randomization and allocation concealment

This investigation strictly follows the framework of randomized controlled trials conducted at a single clinical research facility. A statistician, who is not involved in the study, will use SPSS 22.0 statistical software to generate a sequence of random numbers for the 70 recruited patients, dividing them into two groups: a treatment group that will receive gentle myofascial adjustment therapy and a control group that will undergo traction combined with cervical collar fixation, with each group consisting of 35 participants. The statistician will match the sequence of patient visits to the generated random numbers to finalize the group assignments. These assignments will then be securely stored in opaque envelopes to ensure allocation concealment. During the treatment phase, the physician will access these envelopes in the order of the child's visits to retrieve the group code. The physician will record both the patient code and the group number. Thereafter, after obtaining informed consent and completing the basic information form, the envelopes will be opened in strict numerical order, ensuring that patients receive examinations and treatments as detailed within the envelopes.

### Blinding

Due to the distinct nature of the interventions, blinding of both participants and treating clinicians is not feasible. To maintain methodological rigor, a single-blind approach will be employed: all outcome assessments—including ultrasound measurements, VAS, and NDI scoring—will be conducted by an independent evaluator unaware of the group assignments. Statistical analyses will also be performed by a blinded third-party statistician to ensure impartial interpretation of data.

### Intervention

Cervical traction will be performed with participants in a supine position using a specialized traction apparatus. A cervical traction strap is then obtained for this procedure. The patient lies flat on the bed in a neutral supine posture, and protective cotton gauze is placed under the neck to prevent skin damage during traction. The cervical traction strap is secured beneath the patient's jaw and at the occipital protuberance. We make adjustments to ensure the appropriate tightness while directing traction towards the top of the head. The traction angle, which is the angle between the cervical spine and a horizontal plane during traction, usually ranges from 0° to 15°. The process is beginning with an initial weight will range from 1 to 3 kg, generally not exceeding one-tenth of the patient's body weight, and the specific traction angle and weight are adjusted based on the patient's will be modified according to comfort and the condition throughout the treatment. During traction sessions, the patient's head is stabilized, and family members or nurses are present to closely monitor the patient's condition. If the patient experiences dizziness or discomfort, the nursing staff notify the physician for immediate intervention. Each traction session lasts about 30 min, and is administered twice daily.

### Treatment group

**Figure 3:**
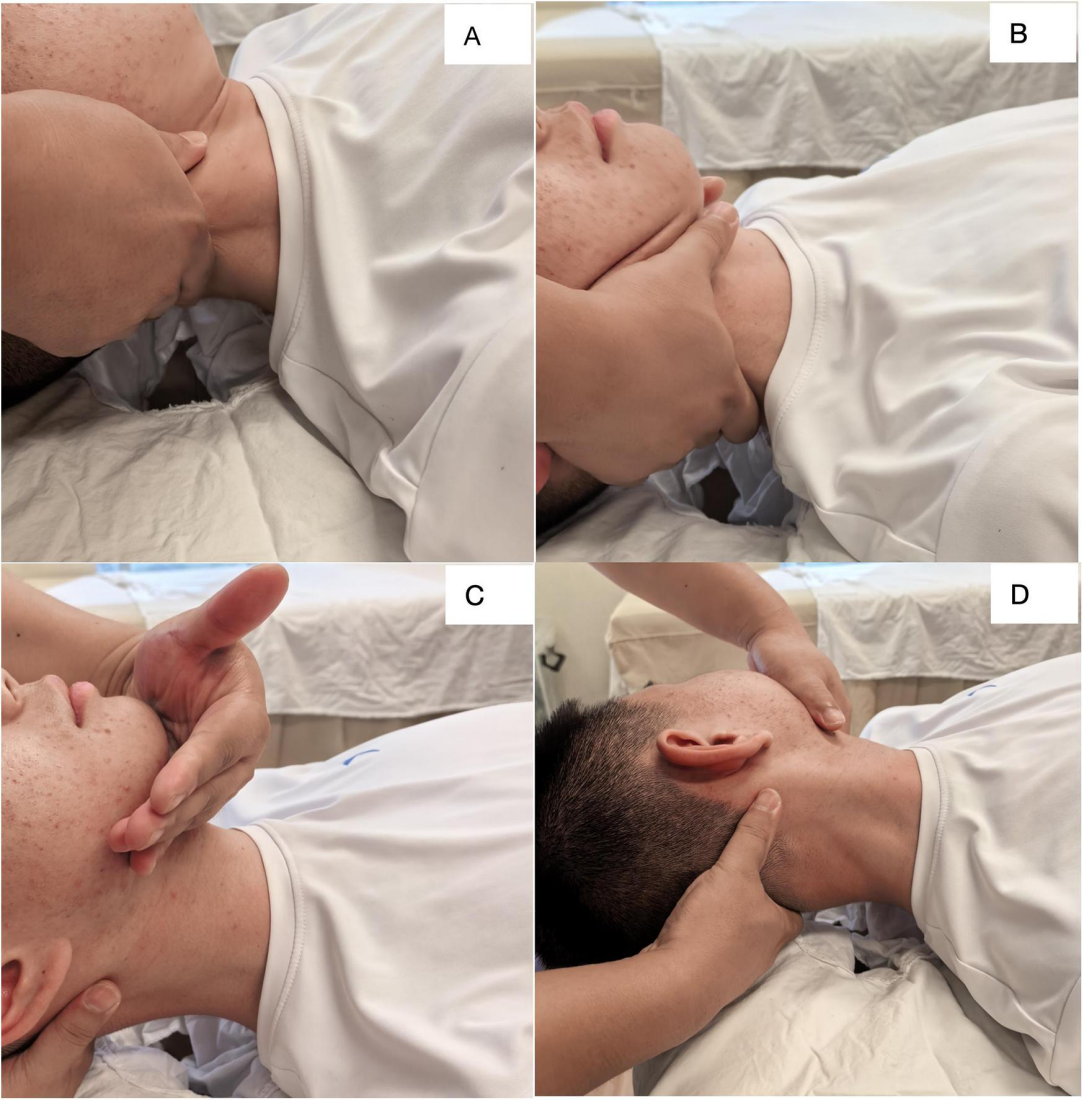
Demonstration of manual techniques. **(A)** Kneading to relax the neck muscles; **(B,C)** micro-adjustment techniques to adjust muscle and skeletal balance; **(D)** Manipulative adjustment of atlantoaxial subluxation.

Gentle muscle adjustment involves the physician sitting or squatting in front of the child's head after cervical traction. Using both hands, they employ various techniques such as pressing, kneading, pinching, and rubbing on the neck muscle groups to relieve muscle tightness and improve neck flexibility, with this process lasting about 10 min. Following this, passive micro-adjustment takes place, where the physician stabilizes both sides of the child's neck with their hands, placing their thumbs on the child's jaw and spreading their fingers across the occipital protuberance. With minimal force, the physician gently pulls upwards towards the top of the head to utilize the traction effect. The child is instructed to cooperate by performing passive neck flexion, extension, lateral bending, and rotation across six specific angles—namely, flexion, extension, left lateral bending, right lateral bending, left rotation, and right rotation, the angle of each movement direction should be approximately 5–10 degrees. During this process, the physician should apply gentle force to the joint capsule with minimal load, ensuring the entire procedure is performed slowly and softly to maintain comfort, lasting approximately five minutes.all done slowly and gently to ensure comfort, lasting around 5 min. Subsequently, during active micro-adjustment, the cervical traction is gradually released, allowing the child to sit on a treatment chair. The physician positions themselves behind the child, placing their thumbs near the GB20 (Gallbladder 20) point at the back of the neck and resting their remaining fingers on either side of the jaw. The physician acupoints and applies light opposing pressure to slightly elevate the cervical spine while guiding the child to actively perform flexion, extension, lateral bending, and rotation, emphasizing movement toward the restricted direction. This stage lasts approximately 10 min. Lastly, emphasizing the importance of muscle relaxation techniques for proper bone alignment, both hands employ gentle techniques, including pressing and kneading, on the child's neck and shoulder muscles. This approach is designed to relax the neck meridians and restore the muscles' physiological properties, and which in turn enhances neck flexibility and ensures the correct anatomical alignment of the atlanto-axial joint, ultimately achieving “bone alignment.” This final treatment phase lasts approximately five minutes and primarily involves gentle pressing and kneading to promote muscle relaxation. The entire treatment regimen is structured to be conducted twice daily, five days a week, over a span of two consecutive weeks. Figure 3 shows the demonstration of manual techniques.

### Control group

After each cervical traction session, a properly sized cervical collar will be securely fastened around the child's neck, along with protective cotton gauze to reduce the risk of skin irritation. The child's head will be kept in a neutral forward position. Measures will be taken to stabilize it and prevent any lateral movements. The length of time the cervical collar is worn will depend on the child's comfort level, with the goal of keeping the collar on for as long as possible, although it will be removed during sleep. If the child experiences any discomfort while wearing the collar, the physician will be notified immediately. The cervical collar will be applied right after each traction session; the treatment plan consists of five sessions per week for two consecutive weeks.

### Outcome measures

#### Primary outcome

The primary measures of interest included muscle thickness that encompasses both the anterior-posterior dimension (APD) and the lateral dimension (LD), along with the Young's modulus value (E value).

##### Musculoskeletal ultrasound examination

The Supersonic Aixplorer ultrasound diagnostic system, situated in the ultrasound department of our hospital, will be utilized for musculoskeletal examinations. This system employs equipped with a 15l linear array probe that operates within a frequency range of 4–15 MHz, making it particularly well-suited for detailed imaging in this area (35).

##### Positioning

To assess the trapezius muscle, the patient should lie in a prone position; with a soft, minimal-thickness U-shaped pillow placed under the forehead. This setup allows the patient's arms placed alongside the head to ensure neutral cervical alignment and full muscle relaxation. For the sternocleidomastoid assessment, patients will be positioned laterally, with a pillow at shoulder- height to maintain a neutral alignment of the head and neck, thereby facilitating full relaxation of the neck and shoulder areas.

##### Measurement location

The anatomical site for measuring the sternocleidomastoid muscle will be taken at the midpoint between the sternal notch and the mastoid process. In the case of the trapezius muscle, the measurement location will begin 2 cm lateral to the C4 spinous process for the trapezius (35).

##### E value measurement

The patient should maintain the prescribed position to ensure complete relaxation of the neck muscles. The examination begins with a standard two-dimensional ultrasound, which involves scanning the muscle tissue longitudinally and subsequently performing a transverse scan while keeping the probe aligned with the muscle fibers. Throughout this process, the ultrasound beam will be positioned orthogonal to the muscle fibers, enabling the visualization of muscle echoes and the arrangement of fibers. Emphasis is placed on achieving a clear view of the target muscle fibers. After this initial scanning, the elastography mode, specifically shear wave elastography (SWE), will be activated to cover the area of interest for the purpose of creating a tissue elasticity map. The system will automatically calculate the elasticity (E) value of the muscle tissue within this designated region of interest. Measurements will be taken three times, and the average values will be recorded.

##### Muscle thickness measurement

The patient will stay in a relaxed position, ensuring that the neck muscles are completely at ease. The examination will begin with a standard two-dimensional ultrasound, starting with a longitudinal scan of the muscle, which keeps the probe's longitudinal axis aligned with the muscle fibers. This will be followed by a transverse scan; during the examination, the ultrasound beam will be kept perpendicular to the muscle fibers, which will help in clearly observing the muscle echoes and the arrangement of the fibers, focusing on obtaining a clear image of the target muscle fibers. The ultrasound device's built-in software will be used to measure the muscle thickness at the defined transverse section. Measurements will be taken three times, and the average of these readings will be recorded.

#### Secondary outcomes

##### Visual analog scale (VAS) for pain

This assessment tool features a visual analog scale that is about 10 centimeters long, divided into 10 increments, with the endpoints labeled “0” and “10.” A score of “0” indicates no pain at all, while a score of “10” represents the highest level of pain imaginable. As the scores increase, they correspond to greater levels of pain intensity, thereby making this scale useful for assessing the severity of neck pain.

##### Neck disability index (NDI)

This commonly used tool evaluates cervical functional status by examining neck pain, related symptoms associated with neck pain, and the capacity to carry out daily activities. It consists of 10 items and has a maximum possible score of 50 points. Higher scores indicate greater functional impairment.

##### Clinical efficacy assessment

The assessment of clinical efficacy will be classified into four categories following the “Standards for Diagnosis and Efficacy of TCM Orthopedic Diseases” and “Standards for Diagnosis and Efficacy of TCM Diseases” that categorize outcomes into four classifications: ① Cure—complete resolution of neck pain, stiffness, and tilt with full cervical mobility; ② Significant Effect: This indicates a notable reduction or improvement in symptoms like neck tilt, neck pain, and muscle stiffness, paired with a considerable enhancement in cervical movement. ③ Effective-moderate symptom improvement and partial restoration of motion; ④ Ineffective: This classification is used when there is no improvement in symptoms, including neck tilt, neck pain, and muscle stiffness, or if there is even a deterioration, with no significant enhancement in cervical movement. The overall efficacy rate will be calculated as: Efficacy rate = (number of cures + number of significant effects + number of effective cases)/total number of participants × 100%.

##### Follow-up observation

Assessment after treatment at the two-week mark, and again during a follow-up observation up evaluations will occur two weeks and six months after completion of treatment to assess symptom recurrence or sustained improvement.

### Statistical methods

Sample size estimation is based on comparisons of two proportions in a fully randomized design following He Yan's work, “Statistics in Traditional Chinese Medicine.” In this framework, the variable p represents the combined rate, while p1 and p2 indicate the known rates for each group. The required sample size for each group is denoted as n. The literature establishes the maximum efficacy rate at 93.5% and the minimum efficacy rate at 73.6%. Assuming equal sample sizes for both groups, *p* can be calculated as the average of *p*1 and *p*2, formulated as *p* = (*p*1 + *p*2)/2, with *q* defined as 1 − *p*. The formula for determining the necessary sample size is *n* = 8*pq*/(*p*1 − *p*2)^2^. Based on this calculation, *n* is estimated to be 28. Considering a projected dropout rate of 15%, the minimum number of observations needed for each group is adjusted to 32 participants to account for dropout, which is derived by dividing 28 by (1–0.15). To further improve the accuracy of the trial data and the resulting statistical outcomes, the sample size will ultimately be increased to 35 participants per group to enhance the reliability of the statistical results.

### Statistical analysis

All data will be analyzed using SPSS version 22.0 software. Categorical data will be summarized as frequencies or percentages and compared using Chi-square tests. Ordinal data will be analyzed using rank-sum tests. For measurement data that follow a normal distribution and demonstrate homogeneity of variance, results will be presented as mean ± standard deviation (x ± s). Independent samples t-tests will be used for between-group comparisons, paired-sample t-tests for within-group comparisons. Non-normally distributed data will be analyzed with nonparametric rank-sum tests. A *P*-value < 0.05 will be considered statistically significant.

## Discussion

**Figure 4:**
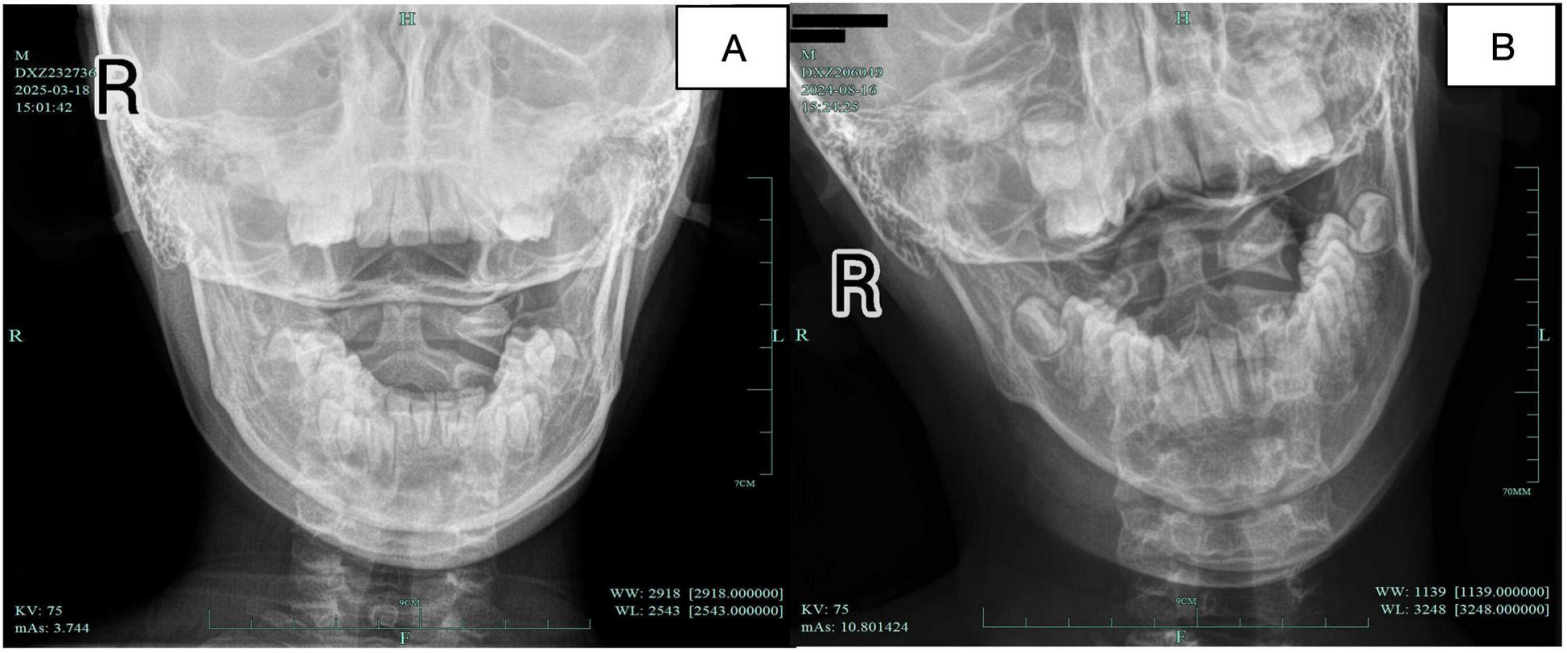
**(A)** Normal atlantoaxial joint x-ray image; **(B)** AARS x-ray image.

The multifactorial causes of atlanto-axial rotatory subluxation (AARS) are a multifactorial condition involving complex biomechanical and pathological mechanisms. Figure 4 shows the normal atlantoaxial joint x-ray image and AARS image. Current treatment options are divided into surgical and conservative approaches (36). Interestingly, traditional Chinese medicine techniques, such as manipulation and bone-setting, have shown clinical effectiveness in treating AARS (37). One notable method is the gentle muscle adjustment technique, which is performed while maintaining continuous traction. This technique is based on the principle of “equal emphasis on muscles and bones, prioritizing muscles.” It creates a localized state of “instability” in the cervical area through traction. The method involves manipulation and dynamic adjustments across six movement axes. These actions restore the normal muscle tone and function, ultimately achieving proper skeletal alignment. The main goal of this approach is to restore the ecological balance between muscles and bones in the atlanto-axial joint. This enhances stability and reduces the risk of recurrence, and thus may provide new insights for the clinical therapy of pediatric AARS.

The current standards for evaluating treatment efficacy primarily depend on subjective assessment scales, which do not provide objective metrics to evaluate treatment effectiveness. The introduction of MUSU technology offers a chance to quantitatively evaluate the effects of the soft tissue manipulation method under continuous traction on cervical muscles, focusing on aspects such as muscle morphology and elasticity (38, 39). This approach aims to investigate the connection between the characteristics of neck muscles and the occurrence of neck pain and functional limitations. Such advancements present a more objective, safe, and cost-effective way to assess clinical outcomes.

However, despite the established effectiveness of the soft tissue manipulation technique combined with continuous traction, research into AARS (Acute Anterior Radial Syndrome) has been in its early stages, marked by significant gaps and a lack of rigorous clinical trials, many of which often rely on anecdotal evidence. Additionally, there is a shortage of evidence-based treatment protocols and randomized controlled trials, which diminishes the reliability of the outcomes reported in existing studies.Our research is based on muscle-bone theory. It involves a randomized controlled trial aimed at assessing the effectiveness of traction along with manipulation and bone-setting for AARS, making it a groundbreaking clinical effort in this field. We expect that the results of this study will provide new insights and approaches for treating conditions related to AARS. Ultimately, we hope that the findings from this trial will help reduce the symptoms experienced by individuals with AARS, lessen their physical and psychological burdens, and improve their overall quality of life.

## Limitations

This study could not be conducted as a fully blinded trial, which introduced bias into the results. We have will highlight the risk of bias and its potential impact in the clinical findings. Future clinical research may benefit from more precise design regarding the implementation of blinding methods. It is worth noting that the musculoskeletal ultrasound examination method mentioned herein also possesses certain limitations. As a relatively novel clinical assessment technique for measuring muscle strength, musculoskeletal ultrasound relies heavily on the operator's technical proficiency and knowledge base. Furthermore, the image quality produced by the ultrasound machine may introduce bias into the muscle measurement results.

## Conclusion

In summary, this study proposes to employ the method of gentle tendon adjustment under continuous traction as the treatment approach, targeting pediatric patients with AARS. A clinical randomized controlled trial methodology will be adopted to observe changes in visual analogue scale (VAS) pain scores, neck disability index (NDI) scores, and alterations in cervical muscle morphology and elasticity before and after treatment. By objectively quantifying clinical efficacy through musculoskeletal ultrasound during sustained traction with gentle tendon manipulation, this study will clarify the clinical value of this approach for treating pediatric AARS, facilitating its future implementation.
